# High-intensity focused ultrasound with visually directed power adjustment for focal treatment of localized prostate cancer: systematic review and meta-analysis

**DOI:** 10.1007/s00345-024-04840-6

**Published:** 2024-03-20

**Authors:** Samuel J. Peretsman, Mark Emberton, Neil Fleshner, Sunao Shoji, Clinton D. Bahler, Larry E. Miller

**Affiliations:** 1 Charlotte, NC USA; 2 Interventional Oncology, Division of Surgery and Interventional Science, University College London, London, UK; 3 Department of Surgical Oncology Urology, Princess Margaret Cancer Centre, University Health Network, Toronto, Canada; 4 Department of Urology, Tokai University School of Medicine, Isehara, Japan; 5 Department of Urology, Indiana University, Indianapolis, IN USA; 6 Miller Scientific, 3101 Browns Mill Road, Ste 6, #311, Johnson City, TN 37604 USA

**Keywords:** Focal therapy, HIFU, High-intensity focused ultrasound, Meta-analysis, Prostate cancer, Systematic review

## Abstract

**Purpose:**

To characterize patient outcomes following visually directed high-intensity focused ultrasound (HIFU) for focal treatment of localized prostate cancer.

**Methods:**

We performed a systematic review of cancer-control outcomes and complication rates among men with localized prostate cancer treated with visually directed focal HIFU. Study outcomes were calculated using a random-effects meta-analysis model.

**Results:**

A total of 8 observational studies with 1,819 patients (median age 67 years; prostate-specific antigen 7.1 mg/ml; prostate volume 36 ml) followed over a median of 24 months were included. The mean prostate-specific antigen nadir following visually directed focal HIFU was 2.2 ng/ml (95% CI 0.9–3.5 ng/ml), achieved after a median of 6 months post-treatment. A clinically significant positive biopsy was identified in 19.8% (95% CI 12.4–28.3%) of cases. Salvage treatment rates were 16.2% (95% CI 9.7–23.8%) for focal- or whole-gland treatment, and 8.6% (95% CI 6.1–11.5%) for whole-gland treatment. Complication rates were 16.7% (95% CI 9.9–24.6%) for de novo erectile dysfunction, 6.2% (95% CI 0.0–19.0%) for urinary retention, 3.0% (95% CI 2.1–3.9%) for urinary tract infection, 1.9% (95% CI 0.1–5.3%) for urinary incontinence, and 0.1% (95% CI 0.0–1.4%) for bowel injury.

**Conclusion:**

Limited evidence from eight observational studies demonstrated that visually directed HIFU for focal treatment of localized prostate cancer was associated with a relatively low risk of complications and acceptable cancer control over medium-term follow-up. Comparative, long-term safety and effectiveness results with visually directed focal HIFU are lacking.

**Supplementary Information:**

The online version contains supplementary material available at 10.1007/s00345-024-04840-6.

## Introduction

Over 1.4 million men worldwide receive a prostate cancer (PCa) diagnosis each year [1], and 1 in 8 men receive this diagnosis during their lifetime [2]. Approximately 87% of these cancers are localized to the prostate without the involvement of nearby organs [3]. While whole-gland tumors are typically treated with radical radiotherapy or prostatectomy, localized tumors may be treated with organ-sparing focal therapies intended to minimize side effects while bridging active surveillance and radical treatment in low- and intermediate-risk patients. High-intensity focused ultrasound (HIFU) is a therapy for PCa that targets energy at the index lesion, resulting in coagulating necrosis of malignant tissue by thermal and mechanical effects while sparing the surrounding non-cancerous prostatic tissue. HIFU is an attractive option for focal therapy of localized tumors, since the lesion with the largest focus of cancer largely determines patient prognosis and metastases risk [4].

HIFU can be classified into algorithm-directed or visually directed treatment protocols. Algorithm-directed HIFU assumes specific tissue-related properties, tissue homogeneity, and fixed ultrasound absorption coefficients that produce thermal ablation using pre-defined power/time combinations at given tissue depths. In contrast, visually directed HIFU allows the user to view prostate tissue changes in real time and make power adjustments to account for natural tissue variability. While several systematic reviews have summarized safety and effectiveness outcomes with HIFU for PCa [5–8], none have reported outcomes of focal therapy with visually directed HIFU. Therefore, the purpose of this systematic review with meta-analysis was to characterize cancer-control outcomes and complications following visually directed HIFU for focal treatment of PCa.

## Methods

The systematic review and meta-analysis followed the Preferred Reporting Items for Systematic Reviews and Meta-analyses (PRISMA) [9]. The review protocol was prospectively registered at http://www.researchregistry.com (reviewregistry1564).

### Study eligibility criteria

Randomized trials and observational studies of visually directed HIFU for focal treatment of PCa were eligible for inclusion in the systematic review. We excluded studies of algorithm-directed HIFU, studies reporting combined results of algorithm- and visually directed HIFU, studies of whole-gland HIFU, studies of salvage HIFU, studies of combination therapy, studies published in abstract form only, review articles or commentaries, studies with insufficient sample size (< 10 patients), studies published in non-English journals, and studies that did not report any outcomes specified in this review.

### Search strategy and study selection process

Two researchers (LM, DF) with experience in systematic review methodology independently searched Medline, Embase, and the Cochrane Central Register of Controlled Trials for potentially eligible studies. The pre-defined search strategies included combinations of diagnosis- and procedure-specific keywords. The Medline search strategy is provided in Supplement Table 1; search strategies for other databases were adapted as necessary. We also manually searched the Directory of Open Access Journals, Google Scholar, and the reference lists of eligible papers and relevant review articles. To account for multiple papers derived from the same primary study or subsamples of the primary study, we preferentially extracted data from the paper with the largest sample size and supplemented missing data using secondary sources as needed. This was an essential element of the review, since previous reviews of HIFU for PCa have included duplicate publications in the analysis. Disagreements related to study eligibility were resolved by discussion. The last search was performed in December 2022.

### Data extraction and outcomes

Data were independently extracted from eligible studies using standardized data collection forms, which included study characteristics, patient characteristics, treatment data, study methodological quality, and main outcomes. Data extraction discrepancies between researchers were resolved by discussion. The methodological quality of eligible studies was evaluated with The National Institute of Health assessment tool applied to before-after studies [10]. Outcomes of this review included prostate-specific antigen (PSA) nadir, the proportion of patients with clinically significant positive biopsy, the proportion of patients receiving whole-gland or focal salvage treatment, the proportion of patients receiving whole-gland salvage treatment, and the prevalence of complications including de novo erectile dysfunction (ED), urinary retention, urinary tract infection, urinary incontinence, and bowel injury.

### Data analysis

We used a random-effects meta-analysis model to calculate a weighted estimate and 95% confidence interval (CI) for each outcome. We estimated heterogeneity among studies with the *I*^2^ statistic where a value of 0% represented no heterogeneity and larger values represented increasing heterogeneity. We evaluated the robustness of the meta-analysis conclusions with a one-study removed sensitivity analysis where the analysis was recalculated following iterative one-at-a-time removal of each study. We performed meta-regressions to identify potential prognostic factors for outcomes reported in at least six studies and with substantial heterogeneity (*I*^2^ > 50%) [11, 12]. The variables of interest included in the meta-regression were patient age, baseline PSA, prostate volume, percentage of patients with extra-prostatic tumor (cT3), percentage of patients receiving neoadjuvant androgen deprivation therapy, median year of treatment, and duration of post-treatment follow-up. Potential publication bias was assessed by visually examining funnel plot symmetry.

## Results

### Study selection

Among 312 papers identified in the literature search, 8 observational studies [13–20] with supplemental data derived from 9 duplicate publications [21–29] were included in the systematic review (Supplement Fig. 1).

### Study characteristics and risk of bias

The review included 1819 unique patients from 5 countries treated with visually directed focal HIFU from 2003 to 2021. The treatment plans for focal HIFU varied widely among studies, ranging from no more than quadrant ablation [20] to urethra-sparing subtotal ablation [18]. The percentage of treated prostate volume was rarely reported. Follow-up duration after visually directed focal HIFU ranged from 6 to 36 months (median 24 months) (Table 1). Among the included studies, the mean patient age ranged from 64 to 72 years (median 67 years), baseline PSA ranged from 5.4 to 8.7 mg/ml (median 7.1 ng/ml), prostate volume ranged from 24 to 46 ml (median 36 ml), the percentage of patients receiving neoadjuvant androgen deprivation therapy ranged from 0 to 27% (median 13%), and most patients were staged as cT1 or cT2 (Table 2). Study quality was rated good for seven studies, fair for one study, and poor for none (Supplement Table 2).

**Table 1:** Study characteristics with focal treatment for localized prostate cancer using visually directed high-intensity focused ultrasound

Primary study	Secondary sources	Subjects	Study location	Years of treatment	Treatment plan	Follow-up (months)
Bass et al. [13]		150	Canada	2013–2017	Focal or hemiablation, few treated with hockey stick template; treatment volume ~ 33% of prostate volume	24
Collins et al. [14]		33	US	2016–2021	Hemiablation	–
Khandwala et al. [15]		73	US	2016–2021	MRI visible tumors plus 8–10 mm margin	13
Muto et al. [16]		29	Japan	2003–2006	Hemiablation, including the peripheral zone of both lobes, preserving urethra	32
Reddy et al. [17]	[21–29]	1379	UK	2005–2020	MRI visible tumors plus at least 5 mm margin, typically leading to quadrant ablation or hemiablation	32
Shoji et al. [18]		45	Japan	2007-	Exclusion of urethra, anterior urethral zone, and one focus in contact with urethral tissue	36
Shoji et al. [19]		90	Japan	2016–2018	Treatment area partitioned by lesion location; treatment volume ~ 49% of prostate volume	21
Yee et al. [20]		20	China	2019–2020	Focal or quadrant ablation	6

*MRI* magnetic resonance imaging, *UK* United Kingdom, *US* United States

**Table 2:** Patient characteristics with focal treatment for localized prostate cancer using visually directed high-intensity focused ultrasound

Primary study	Secondary sources	Age (years)	PSA (ng/ml)	Prostate volume (ml)	Clinical stage			Gleason score^a^	NADT
					cT1	cT2	cT3		
Bass et al. [13]		65	7.7	–	83%	17%	0%	7 (6, 9)	–
Collins et al. [14]		64	6.3	–	0%	100%^b^	0%	7 (6, 8)	–
Khandwala et al. [15]		69	8.1	46	67%	32%	1%	7 (6, 8)	–
Muto et al. [16]		72	5.4	36	86%	14%	0%	6 (4, 10)	24%
Reddy et al. [17]	[21–29]	66	6.9	36	7%	81%	12%	7 (6, 9)	1%
Shoji et al. [18]		64	6.6	32	–	–	–	7 (6, 9)	27%
Shoji et al. [19]		70	7.3	24	0%	100%	0%	6 (6, 8)	0%
Yee et al. [20]		68	8.7	40	–	–	0%	– (6, –)	–

*NADT* neoadjuvant androgen deprivation therapy, *PSA* prostate-specific antigen

^a^Reported as median (min, max)

^b^Pathologic stage 2 (pT2)

### Meta-analysis results

**Fig. 1:**
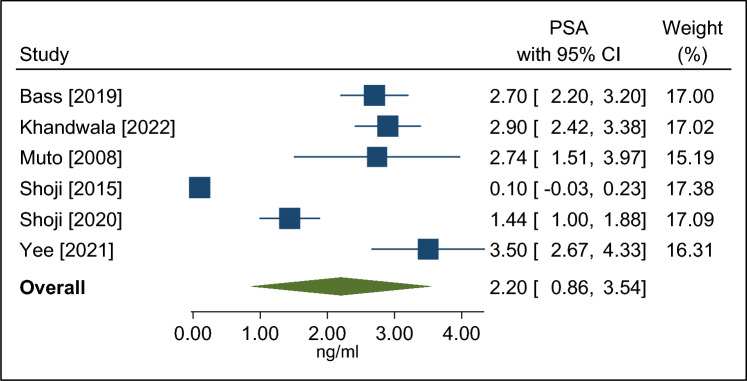
Weighted mean prostate-specific antigen nadir (ng/ml) after focal treatment for localized prostate cancer using visually directed high-intensity focused ultrasound. Mean = 2.2 ng/ml (95% CI 0.9 to 3.5); heterogeneity: *I*^2^ = 98%

**Fig. 2:**
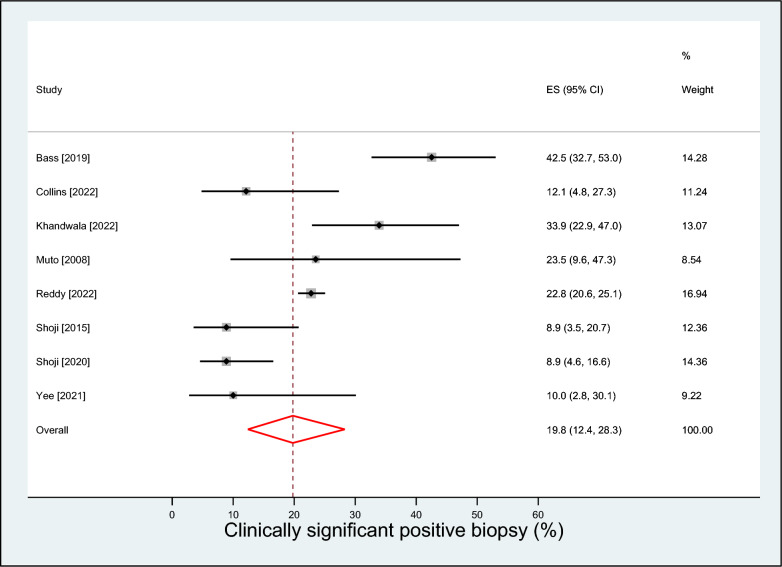
Weighted event rate of clinically significant positive biopsy after focal treatment for localized prostate cancer using visually directed high-intensity focused ultrasound. Event rate = 19.8% (95% CI 12.4–28.3%); heterogeneity: *I*^2^ = 83%

**Fig. 3:**
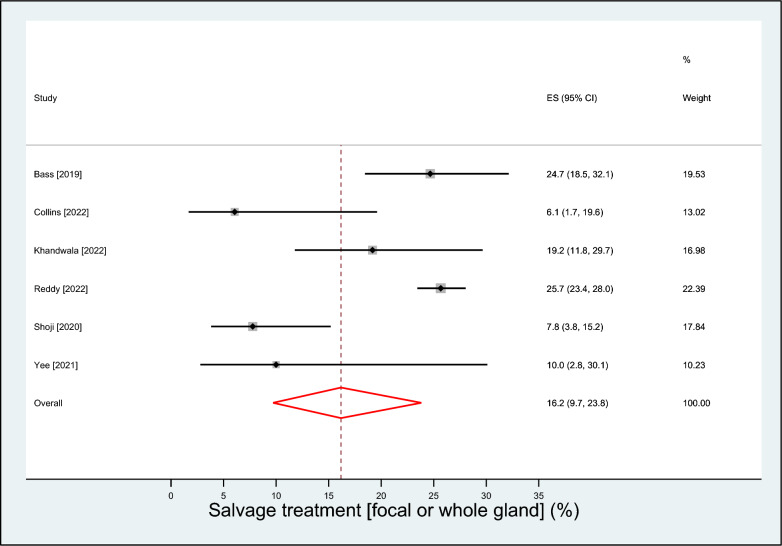
Weighted event rate of focal or whole-gland salvage treatment after focal treatment for localized prostate cancer using visually directed high-intensity focused ultrasound. Event rate = 16.2% (95% CI 9.7–23.8%); heterogeneity: *I*^2^ = 84%

**Fig. 4:**
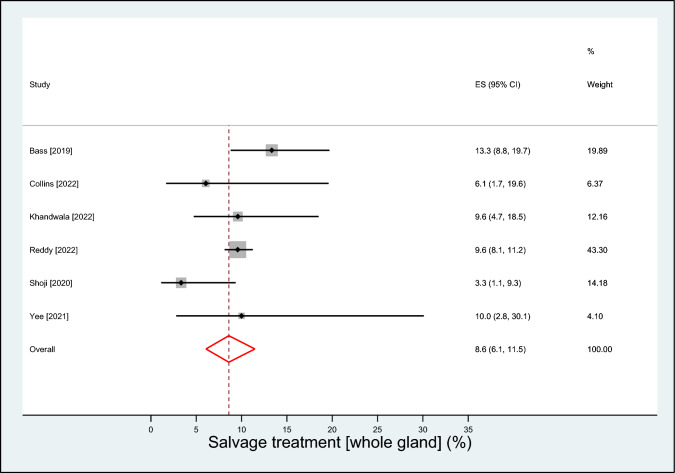
Weighted event rate of whole-gland salvage treatment after focal treatment for localized prostate cancer using visually directed high-intensity focused ultrasound. Event rate = 8.6% (95% CI 6.1–11.5%); heterogeneity: *I*^2^ = 35%

**Fig. 5:**
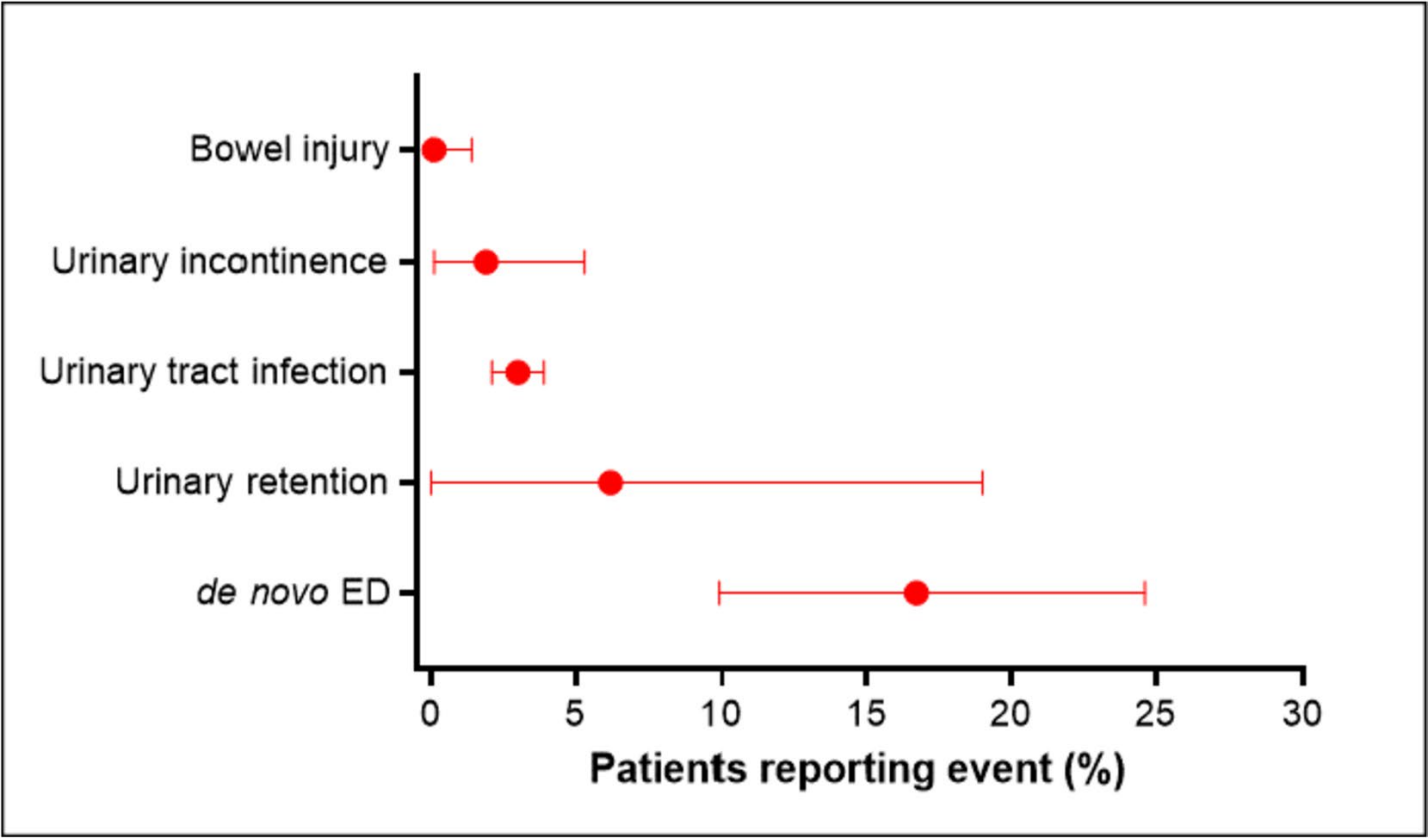
Frequency of complications after focal treatment for localized prostate cancer using visually directed high-intensity focused ultrasound. Plotted values are weighted event rate and 95% confidence interval. *ED* erectile dysfunction

The mean PSA nadir following visually directed focal HIFU varied considerably among studies, ranging from 0.1 to 3.5 ng/ml. The weighted PSA nadir was 2.2 ng/ml (95% CI 0.9–3.5; *I*^2^ = 98%), which was achieved after a median of 6 months post-treatment (Fig. 1). Over a median of 9 month follow-up, a clinically significant positive biopsy was identified in 19.8% (95% CI 12.4–28.3%; *I*^2^ = 83%) of cases (Fig. 2**)**. The rates of salvage treatment were 16.2% (95% CI 9.7–23.8%; *I*^2^ = 84%) for focal- or whole-gland treatment (Fig. 3) and 8.6% (95% CI 6.1–11.5%; *I*^2^ = 35%) for whole-gland treatment (Fig. 4**)**. Complications with visually directed focal HIFU are summarized in Fig. 5. The weighted rates of specific complications were 16.7% (95% CI 9.9–24.6%; *I*^2^ = 63%) for de novo ED (Supplement Fig. 2), 6.2% (95% CI 0.0–19.0%; *I*^2^ = 95%) for urinary retention (Supplement Fig. 3), 3.0% (95% CI 2.1–3.9%; *I*^2^ = 0%) for urinary tract infection (Supplement Fig. 4), 1.9% (95% CI 0.1–5.3%; *I*^2^ = 71%) for urinary incontinence (Supplement Fig. 6), and 0.1% (95% CI 0.0–1.4%; *I*^2^ = 66%) for bowel injury [all rectourethral fistulae] (Supplement Fig. 6).

The meta-analysis results were largely unchanged in the one-study removed sensitivity analyses, suggesting minimal single-study influences on overall outcomes (Supplement Table 3). The meta-regression findings are reported in Supplement Table 4. Larger prostate volume was associated (*p* = 0.002) with a higher clinically significant positive biopsy rate (Supplement Fig. 7), and longer follow-up duration was associated (*p* = 0.007) with lower rates of de novo ED (Supplement Fig. 8). No patient or study characteristic was associated with PSA nadir or the risk of salvage treatment. Funnel plot asymmetry was not evident for any outcome; a formal assessment of publication bias was not performed due to the small number of studies in the review.

## Discussion

Patients with small-volume prostate tumors may be unnecessarily overtreated with whole-gland PCa treatments, which are associated with considerable morbidity [30–32]. Focal treatment approaches are ideal for men with small-volume, single-lobe prostatic tumors who wish to preserve erectile function and continence. We performed the first known meta-analysis of visually directed HIFU for focal treatment of localized PCa. There were several major findings in this meta-analysis of 8 studies comprising 1819 patients treated with visually directed focal HIFU. First, the risks of urinary complications and de novo ED following visually directed focal HIFU were low. Second, visually directed focal HIFU conferred promising cancer-control outcomes with acceptable recurrence rates and 91.4% freedom from whole-gland salvage treatment over mid-term follow-up. Finally, the variability in results among studies included in this meta-analysis was high and only partly explained using meta-regression techniques.

Although there is no consensus for biochemical failure definition after focal therapy [33], a PSA decrease of 70% or greater indicates proper ablation of the index cancer [34–36]. PSA levels after visually directed focal HIFU decreased by approximately 70% among the studies in this review. However, considerable variability was observed in the PSA nadirs in this review (*I*^2^ = 98%). Although we did not identify patient- or study-related factors that influenced the PSA nadir, it is plausible that unmeasured factors, such as the extent and location of malignancy or the volume of ablated tissue, may have contributed to the inconsistency among studies. For example, Yee et al. [20] treated the smallest prostate volume and reported the highest PSA nadir. Conversely, Shoji et al. [18] treated the largest prostate volume and reported the lowest PSA nadir. While objective analysis of this association was not possible due to inadequate reporting of treatment details among studies, a negative association between treated prostate volume and PSA nadir was apparent. Since PSA has poor sensitivity to predict positive biopsies after focal HIFU [37], the clinical importance of these results is unclear.

The weighted rate of clinically significant positive biopsy after visually directed focal HIFU was 19.8%, ranging from 8.9% [18, 19] to 42.5% [13]. This heterogeneity was partially explained in meta-regression where larger prostate volume was associated with higher rates of a clinically significant positive biopsy. While posterior tumors are easily accessible even in larger prostates, HIFU effectiveness in anterior tumors may be limited in larger prostates where energy penetration may be insufficient [26]. Prostate downsizing with neoadjuvant TURP may be considered in patients with larger prostates (> 50 cc) to remove prostatic calcification or abscesses that could attenuate HIFU energy. Unfortunately, the relationship between tumor location and prostate volume was unclear in this review. A second possible reason for the variability in clinically significant positive biopsy rate was that repeat biopsy was performed routinely in some studies, while others reserved re-biopsy only for suspected recurrence or high-risk patients.

An advantage of visually directed HIFU is the ability to make real-time power adjustments based on hyperechoic changes visualized on B-mode ultrasound images. However, only one known study has directly compared the outcomes of visually directed HIFU with algorithm-directed HIFU. In the observational study of Illing et al. [38], men treated with visually directed HIFU for localized PCa achieved statistically lower PSA nadirs than those receiving algorithm-directed HIFU, while rates of urinary complications were numerically lower. Additional support for the potential clinical advantages of visually directed focal HIFU comes from comparing the results of the current review with visually directed focal HIFU to a previous review of focal HIFU in which 67% of studies used algorithm-based HIFU [7]. In that review, the mean PSA nadir ranged from 1.9 to 2.7 ng/ml (vs. 2.2 ng/ml in the current review), the rate of positive biopsy ranged from 14 to 38% (vs. 19.8%), and the incidence of complications was 21% for ED (vs. 16.7%), 11% for urinary tract infection (vs. 3.0%), 9% for retention (vs. 6.2%), and 2% for incontinence (vs. 1.9%). Due to a lack of comparative studies, whether cancer-control outcomes differ between visually directed and algorithm-directed HIFU remains to be determined and warrants further study. Further, no known studies have directly compared the safety or effectiveness of visually directed focal HIFU to active surveillance, radiotherapy, or surgery; thus, any treatment comparisons with visually directed focal HIFU should be considered hypothesis-generating only.

We observed a lower risk of de novo ED in studies with longer follow-up duration. However, only some studies in this review reported temporal trends in ED. In Lovegrove et al. [28], the percentage of men with ED was 10% pre-treatment, increasing to 21% at 1–2 years, and declining to 18% at 2–3 years. In Shoji et al. [18], de novo ED rates decreased during follow-up, from 55% at 3 months, 45% at 6 months, 40% at 1 year, and 37% at 2 years. In Shoji et al. [19], de novo ED rates were 33% at 1 month, 19% at 3 months, 12% at 6 months, 9% at 9 months, and 14% at 1 year. In contrast to these studies, Yee et al. [20] reported increasing ED rates over time, with 0% at baseline, 15% at 3 months, and 30% at 6 months. Overall, most evidence suggests that de novo ED after focal HIFU may be temporary in some men, a finding reported in other reviews [5].

Several limitations pertaining to the quality of the studies included in this review warrant discussion. First, while the high observed heterogeneity in cancer-control outcomes and complications after visually directed focal HIFU afforded the opportunity to explore factors associated with these outcomes, the results of the meta-analysis should be interpreted cautiously. Meta-analysis results are prone to ecological fallacy risks, since inference about individuals is attempted using only study-level information [39]. Additionally, meta-regression is inherently an exploratory analysis considered hypothesis-generating only, and the number of studies available for meta-regression was limited. Consequently, readers are cautioned against drawing causal inferences from the results of this study. Second, the evidence from this review was derived exclusively from observational studies, which have limited internal validity, since they are prone to bias and confounding risks. No clear evidence exists that focal HIFU improves cancer control, quality of life, or comorbidities relative to radiation, surgery, or other focal treatments. Finally, this meta-analysis included results obtained during short- and medium-term follow-up. Although a minimum of 5 years of follow-up was recommended in a Delphi consensus of focal therapies for PCa [33], none of the studies in this review followed patients for this duration. Overall, long-term cancer-control results following visually directed focal HIFU are lacking.

## Conclusion

Limited evidence from eight observational studies demonstrated that visually directed HIFU for focal treatment of localized PCa was associated with a relatively low risk of complications and acceptable cancer control over medium-term follow-up. Future comparative studies with longer term follow-up are warranted to further elucidate the safety and effectiveness of visually directed HIFU for focal treatment of localized PCa.

## Supplementary Information

Below is the link to the electronic supplementary material.

Supplementary file1 (DOCX 740 kb)

## Data Availability

The data from this review will be made available upon reasonable request.
