# Supplementary material for: High-intensity focused ultrasound with visually directed power adjustment for focal treatment of localized prostate cancer: systematic review and meta-analysis
Source: World J Urol. 2024 Mar 20;42(1):175. doi: 10.1007/s00345-024-04840-6 (PMC10954869; doi:10.1007/s00345-024-04840-6)
Supplement: Supplementary file 1 — Supplementary file1 (DOCX 740 kb) [file 345_2024_4840_MOESM1_ESM.docx]

**ONLINE SUPPLEMENT**

**Supplement Table 1. MEDLINE search strategy.^a^**

| Diagnosis Search Terms |
| --- |
| 1. Cancer 2. Carcinoma 3. Neoplasm 4. Prostat* |
| Procedure Search Terms |
| 1. Ablation 2. Focal 3. HIFU 4. High-intensity focused ultrasound 5. Sonablate 6. Transrectal |
| Combination Terms |
| 1. or/1-3 2. 4 3. or/5-10 |
| 1. and/11-13 |

^a^The ‘*’ represents a wildcard symbol used in a search query to represent end truncation.

**Supplement Table 2. Methodological study quality assessment using the National Institute of Health assessment tool for before-after studies.^a^**

| **Study** | **1** | **2** | **3** | **4** | **5** | **6** | **7** | **8** | **9** | **10** | **11** | **12** | **Quality**  **Rating** |
| --- | --- | --- | --- | --- | --- | --- | --- | --- | --- | --- | --- | --- | --- |
| Bass [2019] [13] | Y | Y | Y | Y | Y | Y | Y | N | Y | Y | N | ^b^ | Good |
| Collins [2022] [14] | Y | N | Y | Y | Y | Y | Y | N | N | N | N | ^b^ | Fair |
| Khandwala [2022] [15] | Y | N | Y | Y | Y | Y | Y | N | Y | Y | N | ^b^ | Good |
| Muto [2008] [16] | Y | Y | Y | Y | N | Y | Y | N | Y | Y | N | ^b^ | Good |
| Reddy [2022] [17] | Y | Y | Y | Y | Y | Y | Y | N | Y | Y | N | ^b^ | Good |
| Shoji [2015] [18] | Y | Y | Y | Y | Y | Y | Y | N | Y | Y | N | ^b^ | Good |
| Shoji [2020] [19] | Y | Y | Y | Y | Y | Y | Y | N | Y | Y | N | ^b^ | Good |
| Yee [2021][20] | Y | Y | Y | Y | N | Y | Y | N | Y | Y | N | ^b^ | Good |

N=no; Y=yes.

^a^Item numbers and associated descriptions include: 1) objective clearly stated; (2) eligibility criteria described; (3) representative patient population; (4) all eligible participants enrolled in study; (5) sufficient sample size; (6) intervention described; (7) outcome measures specified; (8) outcome assessors blinded; (9) loss to follow-up and intention-to-treat analysis; (10) statistical analysis of outcome measures before and after intervention; (11) interrupted time-series design; (12) individual data used for group-level effects.

**Supplement Table 3. One-study removed sensitivity analyses of main outcomes of focal treatment for localized prostate cancer using visually-directed high-intensity focused ultrasound.^a^**

| **Outcomes** | **Studies**  **reporting**  **outcome** | **All**  **studies** | **One-study removed,**  **lowest estimate** | **One-study removed,**  **highest estimate** |
| --- | --- | --- | --- | --- |
| PSA nadir (ng/ml) | 6 | 2.2  (0.9-3.5) | 2.0  (0.6-3.4) | 2.6  (1.9-3.4) |
| Clinically significant positive biopsy | 8 | 19.8%  (12.4%-28.3%) | 17.1%  (11.3%-25.1%) | 22.7%  (15.2%-32.4%) |
| Salvage treatment (focal or whole gland) | 6 | 16.2%  (9.7%-23.8%) | 13.9%  (7.9%-23.5%) | 21.7%  (16.6%-27.9%) |
| Salvage treatment (whole gland only) | 6 | 8.6%  (6.1%-11.5%) | 7.7%  (5.3%-10.5%) | 9.1%  (7.7%-10.7%) |
| **Complications** |  |  |  |  |
| *de novo* erectile dysfunction | 6 | 16.7%  (9.9%-24.6%) | 13.3%  (7.0%-24.0%) | 20.2%  (12.9%-30.1%) |
| Urinary retention | 4 | 6.2%  (0.0%-19.0%) | 3.6%  (0.4%-25.4%) | 11.6%  (6.8%-18.9%) |
| Urinary tract infection | 5 | 3.0%  (2.1%-3.9%) | 2.8%  (1.8%-3.9%) | 3.8%  (2.9%-4.9%) |
| Urinary incontinence | 4 | 1.9%  (0.1%-5.3%) | 0.4%  (0.0%-1.8%) | 2.9%  (1.0%-8.0%) |
| Bowel injury | 5 | 0.1%  (0.0%-1.4%) | 0.0%  (0.0%-0.0%) | 0.4%  (0.0%-2.2%) |

PSA=prostate-specific antigen.

^a^Data reported as weighted mean or event rate (95% confidence interval).

**Supplement Table 4. Univariable metaregression of the association of study-level factors with main outcomes after** **focal treatment for localized prostate cancer using visually-directed high-intensity focused ultrasound.^a^**

| **Moderators** | **PSA nadir** | **Clinically significant**  **positive biopsy** | **Salvage treatment**  **(focal or whole gland)** | ***de novo* erectile**  **dysfunction** |
| --- | --- | --- | --- | --- |
| Follow-up duration (mo) | z = -0.07; p = 0.95 | z = 1.08; p = 0.28 | z = 1.32; p = 0.19 | **z = -2.71; p = 0.007** |
| Median procedure year | z = 0.12; p = 0.90 | z = -0.51; p = 0.61 | z = -1.59; p = 0.11 | z = 1.52; p = 0.13 |
| Percent cT3 patients | z = 0.33; p = 0.74 | z = 0.21; p = 0.83 | z = 1.19; p = 0.24 | z = -0.83; p = 0.41 |
| Prostate volume (ml) | z = 1.14; p = 0.25 | **z = 3.12; p = 0.002** | z = 0.87; p = 0.38 | z = 0.43; p = 0.67 |
| Age (years) | z = 0.82; p = 0.41 | z = -0.02; p = 0.99 | z = -1.36; p = 0.17 | z = -0.21; p = 0.83 |
| Pre-HIFU PSA (ng/ml) | z = 1.31; p = 0.19 | z = 0.70; p = 0.49 | z = 0.29; p = 0.77 | z = 0.41; p = 0.68 |
| Percent receiving NADT | z = 1.11; p = 0.27 | z = -0.11; p = 0.91 | z = 0.00; p > 0.99 | z = -0.31; p = 0.76 |

NADT=neoadjuvant androgen deprivation therapy; PSA=prostate-specific antigen.

^a^Positive z-score indicates a positive association with the outcome; a negative z-score indicates a negative association with the outcome. Bolded values indicate statistically significant associations.


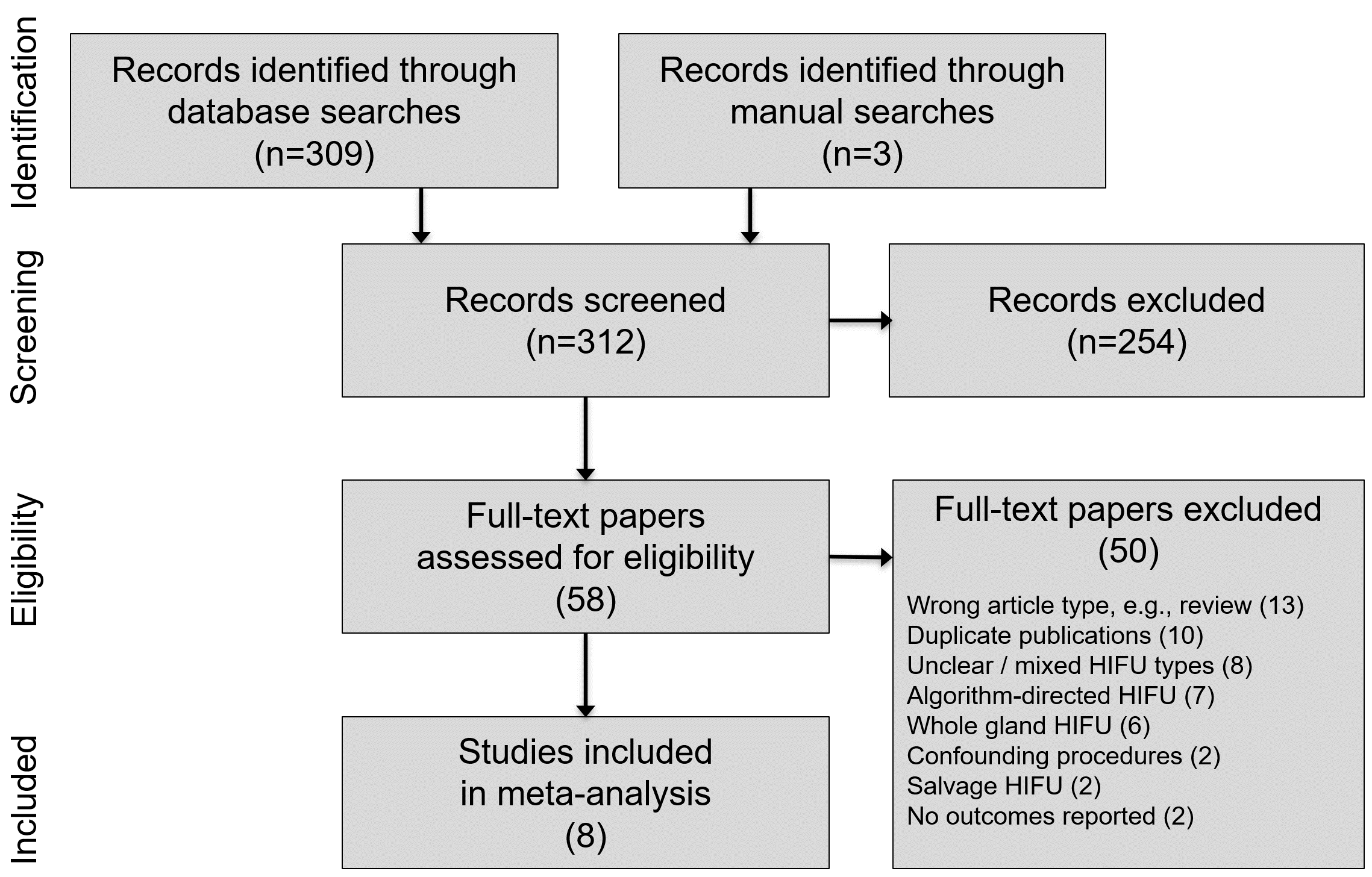

**Supplement Figure 1. PRISMA study flow diagram.**

**Supplement Figure 2. Weighted event rate of *de novo* erectile dysfunction after focal treatment for localized prostate cancer using visually-directed high-intensity focused ultrasound.** Event rate=16.7% (95% CI: 9.9% to 24.6%); Heterogeneity: *I*^2^=63%.

**Supplement Figure 3. Weighted event rate of urinary retention after focal treatment for localized prostate cancer using visually-directed high-intensity focused ultrasound.** Event rate=6.2% (95% CI: 0.0% to 19.0%); Heterogeneity: *I*^2^=95%.

**Supplement Figure 4. Weighted event rate of urinary tract infection after focal treatment for localized prostate cancer using visually-directed high-intensity focused ultrasound.** Event rate=3.0% (95% CI: 2.1% to 3.9%); Heterogeneity: *I*^2^=0%.

**Supplement Figure 5. Weighted event rate of urinary incontinence after focal treatment for localized prostate cancer using visually-directed high-intensity focused ultrasound.** Event rate=1.9% (95% CI: 0.1% to 5.3%); Heterogeneity: *I*^2^=71%.


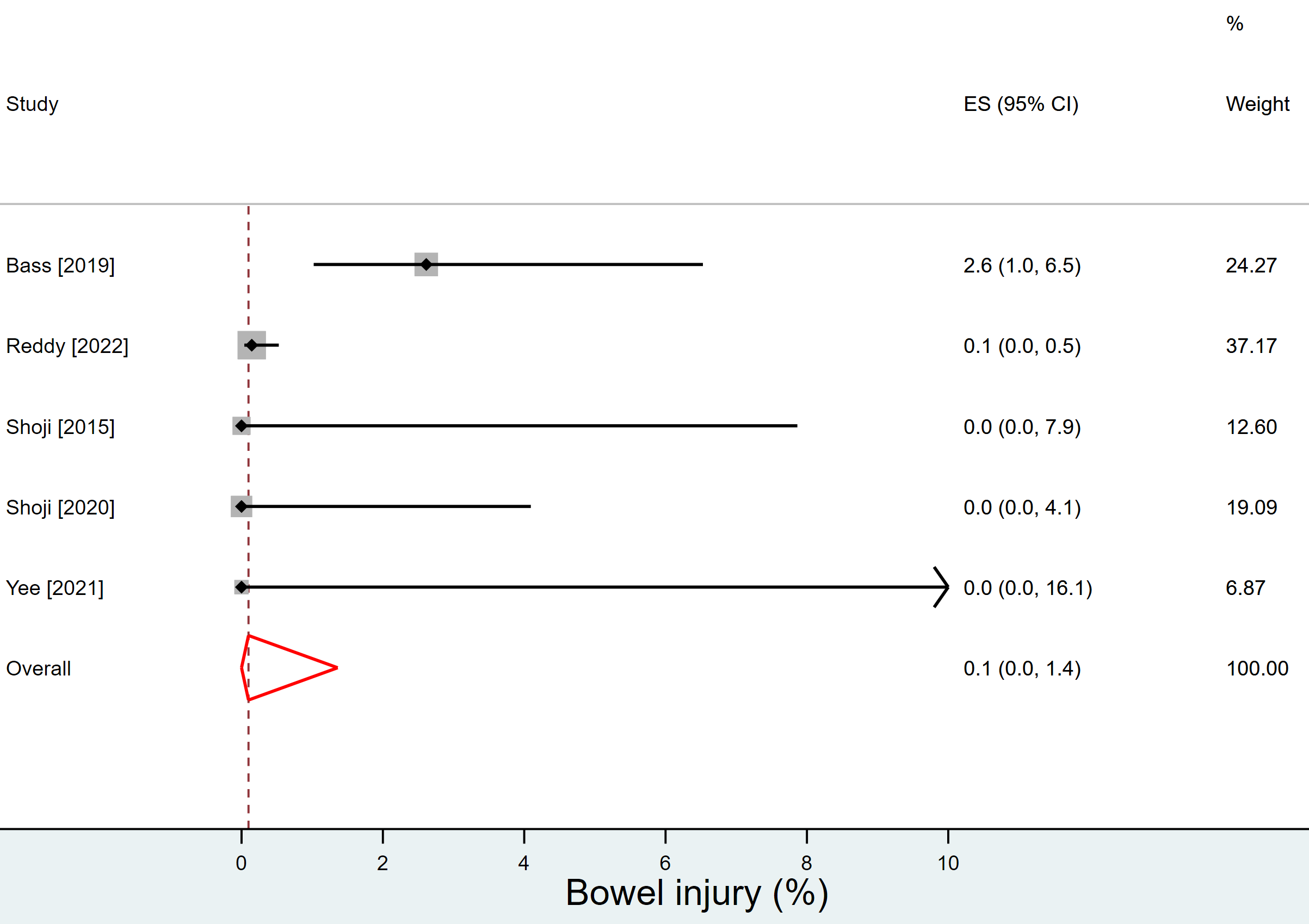


**Supplement Figure 6. Weighted event rate of bowel injury after focal treatment for localized prostate cancer using visually-directed high-intensity focused ultrasound.** Event rate=0.1% (95% CI: 0.0% to 1.4%); Heterogeneity: *I*^2^=66%.


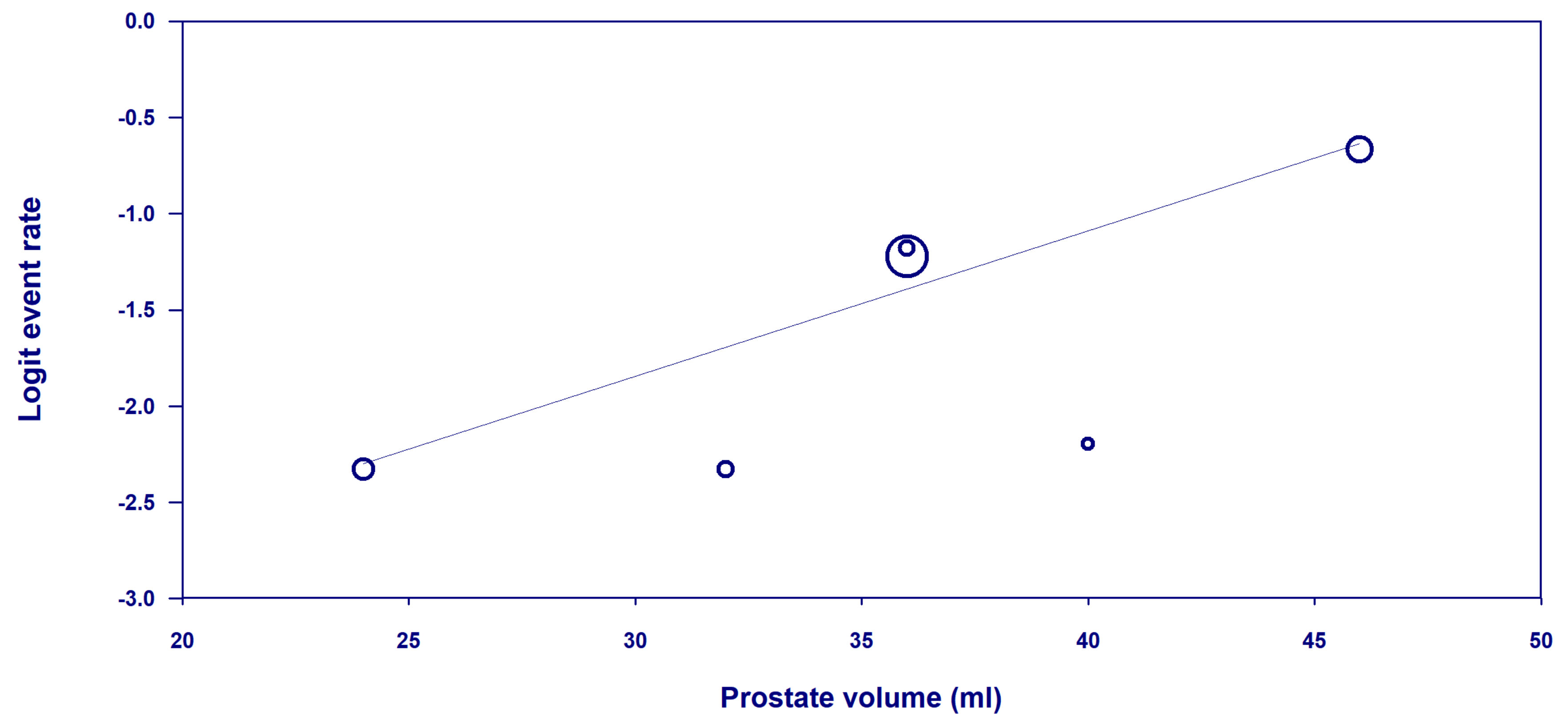


Supplement Figure 7. Bubble plot of the association between prostate volume and the logit event rate of clinically significant positive biopsy after focal treatment for localized prostate cancer using visually-directed high-intensity focused ultrasound. Open circles represent values of individual studies where the circle size is proportional to the study weight in the random-effects model. The line represents the regression line of best fit. P-value for association = 0.002. Logit event rates of 0, -1, -2, and -3 equate to probabilities of 50%, 27%, 12%, and 5%, respectively.


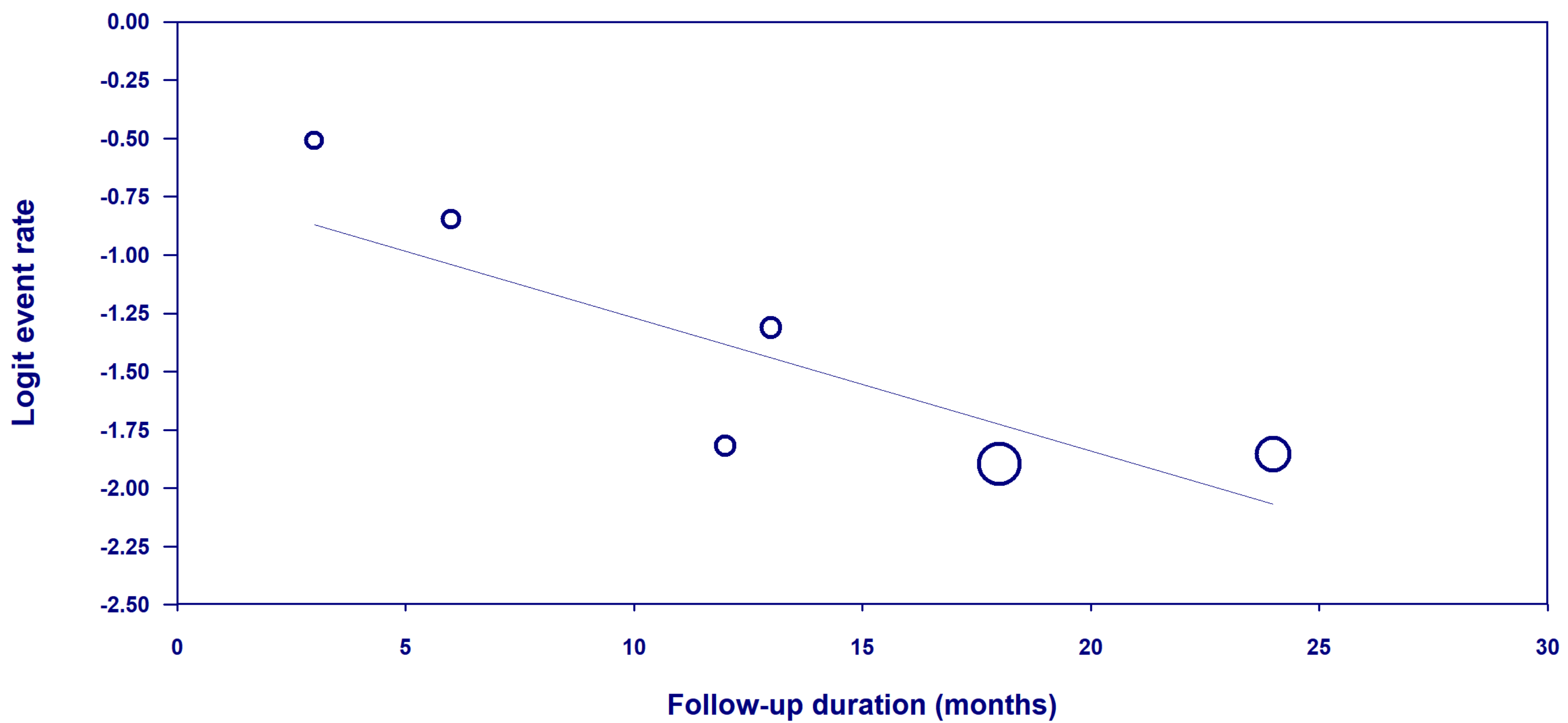


Supplement Figure 8. Bubble plot of the association between follow-up duration and the logit event rate of *de novo* erectile dysfunction after focal treatment for localized prostate cancer using visually-directed high-intensity focused ultrasound. Open circles represent values of individual studies where the circle size is proportional to the study weight in the random-effects model. The line represents the regression line of best fit. P-value for association = 0.007. Logit event rates of 0, -1, and -2 equate to probabilities of 50%, 27%, and 12%, respectively.
